# Genotype-Based Score Test for Association Testing in Families

**DOI:** 10.1007/s12561-015-9128-6

**Published:** 2015-03-17

**Authors:** Hae-Won Uh, Marian Beekman, Ingrid Meulenbelt, Jeanine J. Houwing-Duistermaat

**Affiliations:** 1 Department of Medical Statistics and Bioinformatics, Leiden University Medical Center, S-5-P, P.O. Box 9600, 2300 RC Leiden, The Netherlands; 2 Department of Molecular Epidemiology, Leiden University Medical Center, S-5-P, P.O. Box 9600, 2300 RC Leiden, The Netherlands

**Keywords:** Score test, Multiplex-case and control design, Ascertained familial cases, X-linked SNPs, imputation uncertainty, Incorporation of additional family information

## Abstract

The multiplex-case and control design in which multiple cases are sampled from the same family is considered. In such studies phenotype information of the un-genotyped relatives might be available. We intend to use additional family information when performing genetic association tests. A score test is revisited to provide a flexible framework to accommodate various genetic models and to improve power of the association test by adding available family information. The proposed test accounts for correlations induced by multiple cases from the same pedigree, directly deals with X-linked SNPs in mixed-sex-related samples, and incorporates additional phenotypic information such as the number of (un-genotyped) siblings and parents with similar symptoms by assigning the weights to (genotyped) multiplex cases. In addition, the score test directly incorporates posterior probabilities of imputed genotypes, which leads to an efficiency measure that reflects imputation uncertainty on the test conducted. The proposed test is applied to real applications for illustration. Its efficiency is demonstrated via simulations.

## Introduction

We investigate the use of family-based samples for conducting case–control association analysis. The family-based association tests such as the FBAT [33] are robust against population structure, but lack power because they utilize only the within-family component for the construction of the association test [19, 34, 40]. The population-based tests that incorporate between-family information for family data may be more powerful. A hybrid case–control family-based analysis has been proposed, in which family-based designs integrate unselected controls from other studies into the analysis [21]. In contrast, we incorporate multiplex cases into population-based case–control study in the framework of score test. Although cases as well as controls can be sampled from families, we focus on the multiplex-case and control design using familial cases. The primary advantage of this design would be that familial cases are enriched for genetic factors and therefore may be more informative for genetic research [3]. Such families may have higher frequencies of susceptibility alleles, and the expected difference in frequencies can be greater using multiplex cases and unrelated controls than using independent samples [19, 34]. Since the test statistic to detect association typically depends on the difference in genotype frequencies between cases and controls this design may improve power to detect association, in particular using next-generation sequencing data [48].

When cases are ascertained via multiple affected individuals within pedigrees, the ascertainment issue should be addressed. It is argued that the ascertainment event depends on the phenotype but is conditionally independent of the genotype given disease outcome. The retrospective likelihood, therefore, is appropriate under selection [44, 45]. Using a score test simplifies this matter, in which both prospective and retrospective model can be dealt with [28]. There are other advantages of using a score test based on genotypes. Firstly, it does not require the genotype frequencies to comply with Hardy–Weinberg proportions (HWP). Earlier work by Sasieni [35] demonstrated that statistical tests based on the comparison of allele frequencies rather than genotype frequencies between unrelated cases and controls can have an increased rate of false-positive conclusions when genotype frequencies do not satisfy HWP. Secondly, it provides a simple framework; different dosage scores of the high-risk allele can be used to test for multiplicative, dominant, or recessive effect. Lastly, the score test can be equally applicable for a quantitative phenotype, even when the sample is selected by extremes of phenotype [44].

To consider family-based samples for conducting case–control association analysis, we need to take into account within-family correlations to obtain the proper type I error rates. Generalized estimating equations (GEE) can be used to account for correlations between related individuals [25]. Slager et al. found that this method often fails due to the singularity in the working correlation matrix [37]. Slager and Schaid [36] proposed a statistical test based on the Cochran–Armitage test for trend in proportions [4, 11] which included a variance appropriately accounted for family relationships. Bourgain et al. [5] constructed a quasi-likelihood score (QLS) test statistic that accounts for correlations between individuals by including kinship coefficients, thereby utilizing information from the known pedigree structure. In order to utilize additional phenotype information of un-genotyped family members, Thornton and McPeek [40] proposed the *more powerful* quasi-likelihood score (MQLS) test. This extension of the QLS test incorporates additional phenotypic information of relatives who are not genotyped. That is, the phenotype data of un-genotyped family members are used to give corresponding weights to the (genotyped) multiplex cases. Note that both QLS and MQLS tests are based on the best linear unbiased estimator of allele frequency. These are more suited for related samples from a large complex pedigree for which maximum likelihood estimation is impractical [30]; data collected on the Hutterites and such are outside the scope of this work. Being an allelic test the MQLS test does not have flexibility to test for multiplicative, dominant, or recessive effect. Uh et al. [43] extended the MQLS to genotypic MQLS (gMQLS) test to accommodate different genetic models. Note that the weighting scheme using positive family history, which is similar to that of the MQLS test, has been proposed by Callegaro et al. in allele-sharing statistics for genetic linkage analysis [7]. In this work we incorporate this weighting scheme for the association analysis directly in the score statistic.

Working within the framework of the score test makes other extensions feasible. Firstly, we wish to test for association on the X chromosome in a related sample. For the X chromosome, females have two chromosomes but males have only one. As the X chromosome represents 2.5 % of the human genome for males and 5 % for females, information coming from the X chromosome cannot be ignored. Until recently little research has been reported on performance of such test statistics for association on the X chromosome. For unrelated samples, Loley et al. conducted a broadly conceived simulation study comparing different tests for association on the X chromosome [26]. One option is that after applying an allele-based test to males and females separately the two statistics of $\chi ^2_{(1)}$ distribution can be combined to a test statistic of $\chi ^2_{(2)}$ [46]. Although this approach is straightforward to apply, it often is not a valid test for family data. For example, when a sibling consists of a brother and sister pair, the two $\chi ^2_{(1)}$ tests of males and females are not independent; combining these two statistics becomes rather complicated. Alternatively, for construction of X-linked score test for the multiplex-case and control design, we follow the line of reasoning by Clayton [10]. While males carry 1 copy, in females most loci on the X chromosome are subject to X inactivation [9]; a female will have approximately half her cells with 1 copy active while the remainder of her cells have the other copy activated. In the absence of interaction with other loci or environmental factors, males should be equivalent to homozygous females. Therefore, X loci in males are coded 0 or 2. To account for relatedness in multiplex cases, an X-linked correlation matrix can be calculated either using the ITO matrices of Li and Sacks [22] or using MINX (MERLIN in X, [1]).

In the Genome-Wide Association Studies (GWAS) era, another important point to be considered is the imputation of the genotypes. By borrowing external information of reference haplotypes from the Haplotype Mapping Project (HapMap, http://www.hapmap.org/) and 1000 Genomes Project (http://www.1000genomes.org/), the number of SNPs to be tested increased from 2.5 to 6.7 million [38]. Considering the number of imputed SNPs will increase, and providing computationally efficient software is as important as guarding both accuracy and precision of the test using these imputed genotypes. Therefore, we propose a one-step approach to test and to deal with uncertainty of imputed genotypes. Based on the well-known results concerning the score function for incomplete data [13], we replace the genotype by its posterior expectation given imputed data. The variance of the score statistic measures the statistical information contained in the data. As in Louis [27], Marchini et al. [29] incorporated in the variance term the loss of information from not observing the real genotype. In this manner, the score test provides an efficiency measure $R^2_T$ that reflects the impact of imputed genotypes on the specific test conducted [41, 42]. In order to provide computationally feasible software for dealing with GWAS using imputed SNPs, C++ executable programs (CCassoc and QTassoc) are available at http://www.lumc.nl/uh.

## Methods

### Score Test for the Ascertained Cases

We first address the ascertainment of the independent subjects. Let $Y=(Y_1,\ldots ,Y_n)$ be the phenotype, $X=(X_1,\ldots ,X_n)$ denote genotype dosage 0, 1, or 2. Further, $\bar{Y}$ is the mean of $Y$ in the whole sample, or the proportion of cases in case–control studies. Since the ascertainment event $\mathcal {S}$ depends on the phenotype but is conditionally independent of the genotype given $Y$, $P(X{\,|\,}Y,\mathcal {S})=P(X{\,|\,}Y)$. Therefore, the retrospective likelihood based on $P(X{\,|\,}Y)$ is appropriate under selection [44, 45]. Based on the retrospective likelihood, the score statistic for testing for an additive effect of a genotyped locus on phenotype is

1 $$\begin{aligned} U_X= (Y-\bar{Y})^\top X. \end{aligned}$$

Although the score statistic initially is to model for the effect of genotype on phenotype, the subscript $X$ is to note that $U_X$ is based on the distribution of $P(X{\,|\,}Y)$ [12, 28]. The score statistic $U_X$ is asymptotically normally distributed under $H_0$ with the zero mean and variance

2 $$\begin{aligned} \mathrm{Var}U_X = (Y-\bar{Y})^\top \mathrm{Var}X (Y-\bar{Y}). \end{aligned}$$

Under $H_0$, $U_X^2/ \mathrm{Var}U_X$ is asymptotically distributed as chi-squared with 1 degree of freedom. The variance of genotype $X$ can be estimated by

$$\begin{aligned} \hat{\mathrm{Var}X}=\sigma ^2_X=\tilde{p}_{1}(1-\tilde{p}_{1})+4\tilde{p}_{2}(1-\tilde{p}_{2}) -4\tilde{p}_{1}\tilde{p}_{2}, \end{aligned}$$

where $\tilde{p}=(\tilde{p}_0, \tilde{p}_1,\tilde{p}_2)^\top$ denotes genotype frequency estimate. Here the genotype frequencies do not need to comply with Hardy–Weinberg proportions (HWP). Note that this test can be applied to a binary trait as well as a quantitative phenotype, even when the sample is selected by extremes of phenotype [44]. The score statistic and its variance can be adapted to test other genetic models and for X-linked SNPs.

### Score Test for Related Sample Using Genetic Correlation

When using multiplex cases from the same pedigree, we need to take into account the correlations. We define the correlation matrix $\varvec{K}$ for $n$ subjects as follows:

3 $$\begin{aligned} \varvec{K}&= \begin{pmatrix} 1&{}\quad \rho _{12}&{}\quad \ldots &{}\quad \rho _{1n}\\ \rho _{12}&{}\quad 1&{}\quad \ldots &{}\quad \rho _{2n}\\ \vdots &{}\quad \ldots &{}\quad \ldots &{}\quad \vdots \\ \rho _{1n}&{}\quad \rho _{2n}&{}\quad \ldots &{}\quad 1\\ \end{pmatrix}. \end{aligned}$$

The off-diagonal entries, $\rho _{ij}$s, are twice the kinship coefficient between individuals $i$ and $j$ ($i\ne j$). Here we use prior kinship coefficients [6]; these are based on expected identical by descent (IBD) sharing of randomly chosen alleles from each of the two individuals, and calculated from the pedigree structure.

Let $\Sigma =\sigma _X^2{\mathbf{1}}{\mathbf{1}}^\top$ be $n\times n$ matrix where ${\mathbf{1}}$ represents a vector of ones of length $n$. To take into account correlations induced using multiplex cases from the same pedigree, the expression of the variance of $U_X$ in (2) can be replaced by

4 $$\begin{aligned} \mathrm{Var}U_X = n^{-1} (Y-\bar{Y})^\top [\varvec{K}\circ \Sigma ](Y-\bar{Y}), \end{aligned}$$

where $\circ$ denotes the (Hadamard) termwise product. Because $\Sigma$ is a covariance matrix, it must be symmetric and positive semidefinite and is invertible, and $\varvec{K}$ inherits these properties. Note that $\varvec{K}$ is invertible if and only if the sample does not include both members of any MZ twin pair [40]. Using this modified variance of $U_X$, under $H_0$ the ratio $U_X^2/ \mathrm{Var}U_X$ is chi-squared distributed with 1 degree of freedom [43].

We next describe methods to calculate correlation coefficients of $\varvec{K}$ in (4) for different genetic models and X-linked SNPs to modify the variance of the score test.

### Correlation coefficients for Autosomal Loci

#### Additive Model

To calculate $\rho _{ij}$ in (3), we consider the ITO method [22]. The ITO method uses three stochastic matrices: I, T, and O. Each row of each matrix corresponds to the conditional probability of genotype, given both genotype and IBD status, 2 IBD (I) , 1 IBD (T), and 0 IBD (O), respectively. Let $\rho _T$ be the correlation for the T-component, then the correlation between the $i$th and $j$th relatives for autosomal loci is

5 $$\begin{aligned} \rho _{ij} = \pi _2 + \pi _1 \rho _T \end{aligned}$$

where $\pi _k$ is the probability that the specified relatives should share $k$ alleles IBD. Note that $\rho _{ij}$ is twice the value of kinship coefficient, and $\rho _{ij} =(1/2)^R$ where $R$ is the degree of relationship [39]. For autosomal loci $\rho _T$ equals to 1/2. Hence, for example, the correlations between a sib-pair are $\rho _{\text{ sib-pair }} = 1/4 + 1/2 \rho _T =1/2$, and the correlations between double first cousins are $\rho _{\text{ double-first-cousins }} = 1/16 + 6/16 \rho _T =1/4$. For computation of $\varvec{K}$ existing software such as MERLIN [1] or KinInbcoef [2] can be used. Because we also want to extend the multiplicative models to alternative ones and to test X-linked SNPs, we apply the approach of Li and Sacks. For an outbred population, the correlation matrix consists of sub-blocks of families.

#### Recessive and Dominant Model

For the recessive model the correlation coefficient is no longer independent of allele frequencies. Let $\varvec{K}_{\mathrm{rec}}$ denote the correlation matrix for the recessive model containing $\rho _{\mathrm{rec}ij}$ off the diagonal. We denote $\hat{p}$ the minor allele frequency estimate. The formula for the additive model (5) should be replaced by $\rho _{\mathrm{rec}ij}=\pi _2 + \pi _1 \rho _{T\mathrm{rec}}$, where $\rho _{T\mathrm{rec}}= {\hat{p}}/({1+\hat{p}})$, when random mating condition is satisfied. For example, the correlation of a sib-pair is

$$\begin{aligned} \rho _{\mathrm{rec}{\hbox {sib-pair}}}=\frac{1}{4}+\frac{1}{2}\left( \frac{\hat{p}}{1+\hat{p}}\right) =\frac{1+3\hat{p}}{4(1+\hat{p})}. \end{aligned}$$

The expression in (4) is then $\mathrm{Var}U_X = n^{-1} (Y-\bar{Y})^\top [\varvec{K}_{\mathrm{rec}} \circ \Sigma ](Y-\bar{Y})$ and the element of $\Sigma$, $\sigma _X^2$, can be replaced by $\tilde{p}_{2}(1-\tilde{p}_{2})$, where $\tilde{p}_2$ denotes the frequency of the 2/2 genotype. Analogously assuming complete dominance of one allele over the other allele the above can be applied by flipping the values 1 and 0 for the dominant model.

### Correlation Coefficients for X-linked Loci

An X-linked analog of the autosomal correlation matrix (3) is denoted as $\varvec{K}_X$. For an additive model $\varvec{K}_X$ can be calculated either by the algorithm used in MINX (“MERLIN in X”) [1] or by KinInbcoefX [2]. Deriving the correlation for an X-linked gene is exactly the same as for the autosomal correlation, except that there are four basic correlations, $\rho _T$, in (5). For an additive model we have

$$\begin{aligned} \rho _{T_\mathrm{f,f}}=1/2, \qquad \rho _{T_\mathrm{f,m}}=\rho _{T_\mathrm{m,f}}=1/\sqrt{2}, \qquad \rho _{T_\mathrm{m,m}}=0, \end{aligned}$$

where the subscripts indicate female pairs, mixed pairs, and male pairs, respectively. Thus, the correlation between full sisters with respect to sex-linked traits is

$$\begin{aligned} \rho _{X,\mathrm{sisters}}=1/2+1/2\rho _{T_\mathrm{f,f}}=3/4, \end{aligned}$$

and the correlation for maternal uncle and niece is

$$\begin{aligned} \rho _{X,\mathrm{{maternal-uncle-niece}}}= 1/4\rho _{T_\mathrm{m,f}}=1/4*1/\sqrt{2}. \end{aligned}$$

For females we denote genotypes 0, 1, and 2, and genotypes of males are coded as 0 and 2 by treating males as homozygous females. The expectation of the genotype $X$ is the same in both sexes, while the genotypic variance due to the marker on X chromosome in males ($\sigma ^2_\mathrm{m}$) is twice that in females ($\sigma ^2_\mathrm{f}$). Expected covariances for X-linked are $3/4 \sigma ^2_\mathrm{f}$ for sister pairs, and

$$\begin{aligned} 1/4\rho _{T_\mathrm{m,f}}=1/4*1/\sqrt{2}* \sigma _\mathrm{m}* \sigma _\mathrm{f}=1/4 \sigma ^2_\mathrm{f} \end{aligned}$$

for maternal uncle–niece pair.

The score statistic and its variance can be extended to test recessive effects and for X-linked SNPs using the corresponding correlation matrices.

### Testing for Association at Imputed SNPs

To deal with the uncertainty caused by imputation, we followed the same line of reasoning as in Uh et al. [42] for modifying the score test. Based on the statistical theory for missing data, the genotype data can be partitioned into two parts: $X_\mathrm{comp}=[X_\mathrm{obs}, X_\mathrm{mis}]$ [13, 27]. The log likelihoods for the complete data ($\ell _\mathrm{comp}$) and observed (incomplete) data ($\ell _\mathrm{obs}$) are given by

$$\begin{aligned} \ell _\mathrm{comp} (\theta )&=\log P(X_\mathrm{obs}, X_\mathrm{mis}{\,|\,}\theta ),\\ \ell _\mathrm{obs} (\theta )&=\log \int P(X_\mathrm{obs}, X_\mathrm{mis}{\,|\,}\theta )\mathrm{d}X_\mathrm{mis}. \end{aligned}$$

Let $U(\theta )$ be the complete data score, $\partial \ell _\mathrm{comp} (\theta )/\partial \theta$, and $I(\theta )$ the complete data information, $-\partial \ell _\mathrm{comp}^2 (\theta )/\partial ^2 \theta$, respectively. Instead of observing $X$, for imputed genotypes the posterior probability, $\varpi _i=(\varpi _{i0},\varpi _{i1},\varpi _{i2})$, is given for subject $i = 1,\ldots , n$. Let the expected dosage for the genotype counts of the $i$th individual be

6 $$\begin{aligned} \tilde{X}_i= \mathrm{E}X_i= \varpi _{i1}+2\varpi _{i2}. \end{aligned}$$

Then we replace the genotype counts $X$ by

7 $$\begin{aligned} U_{\tilde{X}}= (Y-\bar{Y})^\top \tilde{X} \end{aligned}$$

in the score statistic (1). As before the score statistic $U_{\tilde{X}}$ is asymptotically normally distributed under $H_0$ with zero mean. Its variance can be determined as in Louis [27]: $\mathrm{Var}(U)= \mathrm{E}(J) -[\mathrm{E}(UU^\top ) - UU^\top ],$ where $J$ is the negative of the second derivative matrix for the complete data and where $\mathrm{E}(J)$ is the expected complete data information over the posterior distribution. The first term of $\mathrm{Var}(U)$ is the variance if there were no “missing data,” and the second term is the penalty for using imputed genotypes. We apply this variance computation to our score $U_{\tilde{X}}$.

Let $\Sigma =\sigma _X^2{\mathbf{1}}{\mathbf{1}}^\top$ be $n\times n$ matrix with the genotypic variance $\sigma _X^2$ where ${\mathbf{1}}$ represents a vector of ones of length $n$. And, the $n\times n$ matrix $\Sigma _\mathrm{loss}$ denotes the loss of information. Then, the score and information for the observed data likelihood are given by

$$\begin{aligned} U_\mathrm{obs} (\theta )&= \mathrm{E}_{X_\mathrm{mis}{\,|\,}X_\mathrm{obs}} U(\theta ),\\ I_\mathrm{obs}(\theta )&= \mathrm{E}_{X_\mathrm{mis}{\,|\,}X_\mathrm{obs}} I(\theta )-\mathrm{Var}_{X_\mathrm{mis}{\,|\,}X_\mathrm{obs}} U(\theta )= \Sigma -\Sigma _\mathrm{loss}. \end{aligned}$$

Here, the term $\mathrm{Var}_{X_\mathrm{mis}{\,|\,}X_\mathrm{obs}} (\cdot )$ represents the loss of information due to imputation uncertainty.

The elements of $\Sigma _\mathrm{loss}$ are defined by the outer product of the square root of individual loss $l_i$,

$$\begin{aligned} l_{i} = \varpi _{i1}(1-\varpi _{i1})+4\varpi _{i2}(1-\varpi _{i2})-4\varpi _{i1}\pi _{i2}. \end{aligned}$$

Thus, on the diagonal we have $\Sigma _{\mathrm{loss}; ii}=l_i$ and off the diagonal we have $\Sigma _{\mathrm{loss}; ij}=\sqrt{l_i l_j}$ for $i, j = 1,\ldots , n$. Then the variance of the score can be expressed as

8 $$\begin{aligned} \mathrm{Var}_{X_\mathrm{obs}} U_{\tilde{X}}=n^{-1} (Y-\bar{Y})^\top \big [\varvec{K}\circ (\Sigma -\Sigma _\mathrm{loss})\big ](Y-\bar{Y}), \end{aligned}$$

where $\circ$ denotes the (Hadamard) termwise product. The relative efficiency measure for case control design of Uh et al. [42] can be used as a quality control measure for accuracy with respect to the association parameter:

9 $$\begin{aligned} R^2_T = \frac{(Y-\bar{Y})^\top [\varvec{K}\circ (\Sigma -\Sigma _\mathrm{loss})] (Y-\bar{Y})}{(Y-\bar{Y})^\top [\varvec{K}\circ \Sigma ]((Y-\bar{Y})}. \end{aligned}$$

Consequently with genotyped data $\Sigma _\mathrm{loss}=0$, hence $R^2_T$ equals to 1. In contrast to the imputation accuracy such as $r^2$ of MACH [24], which is a pre-analysis measure, this post-analysis information measure assigns more weights to associated SNPs [41].

### Incorporation of Family History

Suppose that we have $n+m$ individuals with $n$ and $m$ the number of individuals with non-missing and with missing genotype data at the given marker, respectively. We consider the case–control status of these individuals, and let $Y_n$ and $Y_m$ denote case–control status of genotyped and un-genotyped individuals, respectively. Observe that the MQLS test [40] also treats the genotype $X$ as a random variable, and that the phenotype and family relationship are treated as the weight. The cases get a weight of 1 and population controls get weight of 0. Let $\varvec{K}_{n, m}$ denote $n\times m$ matrix that give correlations between non-missing and missing individuals. Then we propose the new weight for the score test as

10 $$\begin{aligned} Y^*=Y_n+\varvec{K}_{n,m}Y_m. \end{aligned}$$

For example, when a case has two un-genotyped affected siblings, the weight is

$$\begin{aligned} Y^*=1+(1/2 \,\, 1/2) \left( \begin{array}{c} 1 \\ 1 \end{array} \right) =2 \end{aligned}$$

Note that this weight is based only on phenotype and genetic relationship. Therefore, we can replace $Y$ with the corresponding new weight $Y^*$ in the score statistic

11 $$\begin{aligned} U^*_X&=(Y^*-\bar{Y}^*)^\top X, \end{aligned}$$

and in its variancs

$$\begin{aligned} \mathrm{Var}U^*_X =n^{-1} (Y^*-\bar{Y}^*)^\top [\varvec{K}\circ \Sigma ](Y^*-\bar{Y}^*). \end{aligned}$$

## Simulations

To evaluate the performance of the test statistics, the following ascertainment schemes were considered: one affected and two unaffected (ASP1), two affected and one unaffected (ASP2), and three affected siblings (ASP3). For testing we select 1 genotyped individual from each family as cases and use the score test, which is the Cochran-Armitage test for trend in proportions [4, 11]. In addition, we explore whether the power increases by including phenotypic information of un-genotyped relatives. When the type of families is uniform in all families—for example in ASP2 each family consists of 1 affected genotyped sibling and 2 additional phenotype of un-genotyped siblings, the both score tests with and without family information are equivalent. Therefore, we mix the three types of datasets (Mixed): 200 cases are selected from each of the 3 types of ascertainment schemes (ASP1, ASP2, and ASP3). In particular, we implemented the following simulation procedure:

- For case population, parental genotypes are generated assuming random mating and Hardy–Weinberg equilibrium for minor allele frequencies of 0.01, 0.05, 0.07, 0.10, and 0.30.
- Conditional on parental genotypes we generate genotypes of 3 offspring assuming Mendelian transmission.
- Using the logistic model, disease indicators for offspring were generated. The model we considered was
  12 $$\begin{aligned} \mathrm{logit}(\mu _{i})=\beta _0+\beta _g x_{ig}, \end{aligned}$$
  where $x_{ig}$ is the genotypic score for a diallelic gene. The parameter $\beta _0$ denotes the intercept and was determined by $\beta _0=\mathrm{logit}(K_b)$, where $K_b=10~\%$, the baseline disease risk under $H_0$, is used. For evaluation of type 1 error rate, we simulate data under the null, so that the odds ratio, $\exp (\beta _g)$, was set to 1. For simulating data under alternative hypothesis the genotype effect in the logistic regression model in Eq. (12) was modeled as follows: $\exp (\beta _g)$, the odds ratio, was equal to 1.2 and 1.5.
- Since we are dealing with a complex phenotype we also included some residual familial correlation due to the polygenic or environmental sources in our disease model. The broad sense heritability can be written as $h^2\sim \sigma _u^2/(\sigma _u^2+\sigma _E^2)$, where the residual error term $\sigma _E^2$ represents the non-genetic residual familial correlations. This $E$ part quantifies components of variance on the logit scale rather than on the original scale of the (underlying) phenotype, $\sigma _E^2$ can be approximated as $\exp (1)\approx 3$ assuming $E\sim N(0,1)$. Consequently following the error distribution $N(0, \sigma _u^2)$ and by setting $\sigma _u^2$ equal to 1, we obtained the broad sense heritability $h^2$ equal to 25 %. Similarly, by setting $\sigma _u^2=0.5$ , we obtained $h^2$ equal to 14 %.
- For control population the steps (a) and (b) are performed with exception that in the step (b) only the genotypes of one individual are generated.
- Each sampling experiment under additive and recessive genetic model and various ascertainment schemes consisted of 1000 independent replicates: 500 cases 500 controls for the ascertainment schemes ASP1, ASP2, ASP3, and 600 cases and 600 controls for the Mixed ascertainment.
- For each replicate, we tested for an association using (i) the score test (ST) and (ii) where appropriate the score test that includes phenotypic information of un-genotyped relatives (ST$_\text {fam}$).

For generating X-linked genotypes in the step 1, maternal genotypes are generated as above. For paternal genotypes, only one allele is generated, which follows the Bernoulli distribution; the other allele is fixed as Y, which contributes to determine the gender of offspring in the step 2.

### Simulation Results

Type I error rates are reported for each statistic, where the type I error rate is the proportion of significant replicates at the nominal level of 5 % out of the total number of replicates. With 1000 replicates and a true type I error rate of 5 %, 95 % of the empirical estimates are expected to fall between 0.036 and 0.064. In Table 1 the results from the simulation are shown. The baseline prevalence rate has been assumed to be 10 %. For the additive model the type I error rate of the score test under the ascertainment schemes ASP1 and ASP2 was within the expected range of (0.036, 0.064), except the type 1 error rate of 0.35 under the ascertainment scheme of ASP3, 1 selected from 3 affected siblings. For the Mixed ascertainment, both tests showed appropriate type 1 error rate. For the recessive model, under each ascertainment scheme the type 1 error rate of the both tests was too conservative to test rare variants with MAF 5 %, or the corresponding frequency of the homozygotes 0.25 %.

**Table 1:** Empirical type I error rates: calculated by the proportion of significant replicates at the nominal level of 5 % out of 1000 simulations

Model	Ascertainment	Tests	Case/control	Under H0				
Additive				Minor allele frequency (MAF)				
				0.01	0.05	0.07	0.1	0.3
	ASP1	ST$^\mathrm{a}$	500/500	0.050	0.050	0.046	0.046	0.053
	ASP2			0.050	0.052	0.04	0.056	0.048
	ASP3			0.049	0.035	0.049	0.036	0.035
	Mixed	ST	600/600	0.050	0.046	0.052	0.049	0.049
		ST${_\mathrm{fam}}^\mathrm{b}$		0.044	0.044	0.047	0.049	0.045

Recessive				Frequency of homozygote$^\mathrm{c}$				
					0.0025	0.0049	0.010	0.090
	ASP1	ST	500/500		0.029	0.054	0.048	0.049
	ASP2				0.020	0.045	0.052	0.050
	ASP3				0.018	0.038	0.036	0.038
	Mixed	ST	600/600		0.027	0.051	0.049	0.048
		ST$_\mathrm{fam}$			0.028	0.040	0.054	0.048

$$^\mathrm{a}$$ Score test

$^\mathrm{b}$ Score test including phenotypic information of un-genotyped relatives

$^\mathrm{c}$ Equal to square of MAF

Table 2 presents the results of power analysis: the score test (ST) and the score test including positive family history (ST$_\mathrm{fam}$). We simulated the same models as those found in Table 1, namely the same ascertainment schemes discussed previously. Since no statistic examined in Table 1 showed the inflated type I error rates, the comparisons of the estimated power for all simulated models are valid. The power was calculated as the proportion of significant replicates at the nominal level of 5 % out of the total number of replicates. From Table 2, in general selecting cases with positive family history is advantageous; power increased by selecting cases from multiple affected siblings. For the additive model, with weaker association (OR = 1.2) the gain in power was greater when residual variance was small ($h^2=14~\%$). For the recessive model for rare variants (frequency of homozygote ${\le } 0.01$) there was little power to detect association, whereas the gain in power was remarkable under the ascertainment schemes that required a minimum of two affected individuals in each family. Table 2 also presents power results from the Mixed ascertainment scheme of 600 cases and 600 controls, in which each 1/3 of cases were selected from ASP1, ASP2, and ASP3 families. For the rare homozygotes, type 1 error rate was too conservative to test the recessive effects. Overall the ST$_\mathrm{fam}$ test was more powerful than the ST test.

**Table 2:** Empirical power for autosomal SNPs: calculated as the proportion of significant replicates at the nominal level of 5 % out of the total number of replicates

Model	h2	Ascertainment	ST	Case/control	OR = 1.2					OR = 1.5				
Additive														
					Minor allele frequency (MAF)									
					0.01	0.05	0.07	0.1	0.3	0.01	0.05	0.07	0.1	0.3
	25 %	ASP1	ST$^\mathrm{a}$	500/500	0.066	0.080	0.093	0.139	0.240	0.092	0.244	0.307	0.383	0.727
		ASP2			0.072	0.134	0.163	0.217	0.431	0.126	0.440	0.630	0.722	0.954
		ASP3			0.086	0.187	0.254	0.328	0.680	0.171	0.709	0.755	0.914	0.999
		Mixed	ST	600/600	0.078	0.118	0.182	0.226	0.522	0.147	0.545	0.653	0.761	0.981
		ST${_\mathrm{fam}}^\mathrm{b}$			0.082	0.149	0.212	0.287	0.558	0.136	0.579	0.695	0.837	0.992
	14 %	ASP1	ST	500/500	0.049	0.115	0.117	0.157	0.301	0.039	0.127	0.218	0.325	0.624
		ASP2			0.097	0.257	0.209	0.355	0.568	0.086	0.519	0.59	0.833	0.984
		ASP3			0.105	0.284	0.714	0.76	0.772	0.112	0.824	0.969	0.986	1
		Mixed	ST	600/600	0.090	0.242	0.386	0.453	0.622	0.245	0.808	0.908	0.963	0.999
		ST$_\mathrm{fam}$			0.095	0.259	0.520	0.555	0.664	0.312	0.877	0.959	0.984	1

Recessive														
					Frequency of homozygote$^\mathrm{c}$									
					0.0025	0.0049	0.01	0.09	0.0025	0.0049	0.01	0.09		
	25 %	ASP1	ST	500/500	0.032	0.043	0.063	0.139	0.001	0.006	0.025	0.185		
		ASP2			0.020	0.048	0.069	0.221	0.002	0.014	0.066	0.482		

Model	h2	Ascertainment	ST	Case/control	OR = 1.2					OR = 1.5				
		ASP3			0.024	0.064	0.08	0.392	0.004	0.032	0.126	0.81		
		Mixed	ST	600/600	0.045	0.060	0.082	0.264	0.088	0.107	0.193	0.795		
			ST$_\mathrm{fam}$		.031	0.061	0.077	0.307	0.085	0.161	0.253	0.833		
	14 %	ASP1	ST	500/500	0.029	0.049	0.057	0.179	0.002	0.012	0.034	0.256		
		ASP2			0.059	0.077	0.115	0.294	0.010	0.019	0.136	0.800		
		ASP3			0.034	0.178	0.209	0.607	0.018	0.084	0.374	0.996		
		Mixed	ST	600/600	0.046	0.101	0.131	0.419	0.129	0.178	0.44	0.974		
			ST$_\mathrm{fam}$		0.051	0.153	0.159	0.452	0.167	0.238	0.559	0.995		

$$^\mathrm{a}$$ Score test

$^\mathrm{b}$ Score test including phenotypic information of un-genotyped relatives

$^\mathrm{c}$ Equal to square of MAF

Table 3 shows the results of power analysis for X-linked SNPs. Overall the results show the similar findings as for autosomal SNPs. While in case of rare variants (MAF = 0.01) our proposed tests (ST and ST$_\mathrm{fam}$) have little power; for more common variants the gain in power can be observed.

**Table 3:** Empirical power for X-linked SNPs: calculated by the proportion of significant replicates at the nominal level of 5 % out of 1000 simulations

Model	h2	Ascertainment	ST	Case/control	OR = 1.2					OR = 1.5				
Additive														
					Minor allele frequency (MAF)									
					0.01	0.05	0.07	0.1	0.3	0.01	0.05	0.07	0.1	0.3
	25 %	ASP1	ST$^\mathrm{a}$	500/500	0.021	0.039	0.073	0.108	0.280	0.064	0.342	0.418	0.598	0.901
		ASP2			0.023	0.068	0.163	0.217	0.431	0.126	0.440	0.630	0.722	0.954
		ASP3			0.037	0.163	0.222	0.198	0.576	0.166	0.750	0.851	0.940	1.000
		Mixed	ST	600/600	0.019	0.072	0.118	0.133	0.321	0.089	0.402	0.554	0.693	0.944
			ST${_\mathrm{fam}}^\mathrm{b}$		0.045	0.086	0.180	0.158	0.412	0.141	0.559	0.710	0.821	0.986
	14 %	ASP1	ST	500/500	0.011	0.034	0.042	0.052	0.107	0.032	0.107	0.164	0.195	0.440
		ASP2			0.033	0.110	0.164	0.237	0.544	0.127	0.707	0.830	0.907	0.997
		ASP3			0.025	0.387	0.474	0.572	0.952	0.315	0.994	0.9979	1.000	1.000
		Mixed	ST	600/600	0.021	0.151	0.224	0.292	0.646	0.126	0.860	0.909	0.967	1.000
			ST$_\mathrm{fam}$		0.026	0.212	0.325	0.403	0.807	0.228	0.957	0.974	0.995	1.000

$$^\mathrm{a}$$ Score test

$^\mathrm{b}$ Score test including phenotypic information of un-genotyped relatives

## Applications to Real Data

### The Leiden Longevity Study (LLS)

For association of X-linked SNPs and illustration of using imputed probabilities of the genotypes, we apply the proposed test to data from the Leiden Longevity Study (LLS) [17, 41].

In the Leiden Longevity Study (LLS), long-lived families are investigated for parameters contributing to the longevity phenotype. Families were included if at least two long-lived siblings were alive and fulfilled the age criterion of 89 years or older for men and 91 years or older for women. In total, 944 long-lived proband siblings were included with a mean age of 94 years (range 89–104), 1671 offspring (mean age 61, range 39–81), and 744 partners (mean age 60, 36–79). Nonagenarian siblings were genotyped using Illumina660W (Rotterdam, Netherlands) and their partners were genotyped using Illumina660W or OmniExpress (Estonina Biocentre, Genotyping Core Facilty, Estonina). GenomeStudio was used for genotyping calling algorithm. Sample call rate was ${>}95~\%$, and SNP exclusions criteria were Hardy–Weinberg equilibrium $p$ value ${<} 10^{-4}$, SNP call rate ${<}95~\%$, and minor allele frequency ${<} 1~\%$. The number of the overlapping SNPs that passed quality controls in both samples was 296 K. To increase the overall coverage of the genome to 2.5 million SNPs, we imputed autosomal SNPs with HapMap (Haplotype Mapping Project, http://www.hapmap.org) release 22, build 36 of the CEU sample. The imputation program IMPUTE2 (http://mathgen.stats.ox.ac.uk/impute/impute_v2.html) was used.

For association of X-linked SNPs, we consider an affected sibling pair (ASP) and control design: 933 long-lived subjects from 420 nonagenarian sibling pairs are served as cases and 741 partners of offspring as controls. Additional phenotypic information (current age or age at death) for un-genotyped (or deceased) family members (parents or additional siblings) was included: 404 long-lived relatives. Here, to take into account different expectancies in the different birth cohorts, we used life tables for the Dutch population to define the longevity phenotype, the top 10 % of the specific birth cohorts [17]. In Table 4 the results are shown using an arbitrary threshold of $p$ value ${<}0.0001$. From ca. 5900 SNPs on X chromosome tested, no SNP was found to be genome-wide significant. To determine approximate genome-wide significance thresholds as described in [16], the ratio of effective and actual number of tests (0.2) was used to estimate the number of the tests and to correct for multiple testing, namely $p$ value = 1E$-$05. Only rs12840872 exceeded the corrected significance level (*p* value = 8.47E$-$06). Further ST$_\mathrm{fam}$ test that incorporates positive family history gave smaller $p$ values, than the ST test.

**Table 4:** Results of association testing for the X-linked SNPs

Marker	Position	al1	al2	$N{_0}^\mathrm{a}$	$N{_1}^\mathrm{a}$	EAF${_0}^\mathrm{b}$	EAF${_1}^\mathrm{b}$	ST$^\mathrm{c}$	ST${_\mathrm{fam}}^\mathrm{d}$
**rs12840872**	X:4942237	T	C	741	930	0.458	0.510	**8.08E** $-$ **06**	**1.51E** $-$ **07**
rs11094824	X 22212620	A	G	741	925	0.452	0.491	1.64E$-$02	2.86E$-$04
rs10855652	X:86416874	G	A	741	933	0.443	0.488	1.18E$-$02	5.51E$-$04

Bold numbers indicate approximate genome-wide significance

$^\mathrm{a}$ Number of controls and cases

$^\mathrm{b}$ Allele frequency of al2 in controls and cases

$$^\mathrm{c}$$ Score test

$^\mathrm{d}$ Score test including positive family history

Imputing SNPs that are not directly genotyped but are present on a reference panel such as the HapMap usually results in not one imputed value but three probabilities of the possible genotype value, 0, 1, or 2 for each individual. To deal with genotype uncertainty, one can choose the “best” genotype—genotype with the largest posterior probability, or one can use expected genotype counts (genotype dosages) as in (6). By calculating the variance of the score, there are again two options: incorporating uncertainty as in our method, or ignoring uncertainty, which is equivalent to setting $\Sigma _\mathrm{loss}=0$ in (8). We consider three scenarios: (i) “best” guess of genotype and $\Sigma _\mathrm{loss}=0$, (ii) genotype dosage and $\Sigma _\mathrm{loss}=0$, and (iii) genotype dosage and incorporating imputation uncertainty, $\Sigma _\mathrm{loss}\ge 0$ (8). For extensive simulation studies comparing these three approaches that deal with uncertainty in analysis of imputed genotypes, we refer to [20, 47].

For illustration purpose, we chose an extreme case: a SNP, rs17183864 (chromosome 6), with MAF 1 % and the post-imputation measure $r^2=0.3$ of MACH [24] or *info* of IMPUTE, which is the default threshold for ensuring imputation quality. As shown in Table 5, selecting the most likely genotype performs poorly, even though this specific SNP passed the pre-set imputation accuracy threshold, $r^2=0.3$. The results also shows that incorporating imputation uncertainty can be a more powerful method for testing imputed SNPs with corresponding post-analysis quality measure $R_T^2=0.78$ in (9).

**Table 5:** Comparison of methods to deal with uncertainty caused by genotype imputation

	$U{_{\tilde{X}}}^\mathrm{a}$	$\mathrm{Var}_{X_{obs}} U{_{\tilde{X}}}^\mathrm{b}$	ST	$R_T^2 {}^\mathrm{c}$
(i) “best” guess & $\Sigma _\mathrm{loss}=0^\mathrm{d}$	3.24	219.71	4.66E$-$01	1.00
(ii) genotype dosage & $\Sigma _\mathrm{loss}=0^\mathrm{d}$	20.64	18.6	1.71E$-$06	1.00
(iii) genotype dosage & $\Sigma _\mathrm{loss}\ge 0$	20.64	16.53	3.87E$-$07	0.78

$$^\mathrm{a}$$ Equation (7)

$$^\mathrm{b}$$ Equation (8)

$^\mathrm{c}$ Post-analysis information measure as in Eq. (9)

$$^\mathrm{d}$$ Ignoring uncertainty

### The Genetics, Arthrosis, and Progression (GARP) Study

The Genetics osteoARthritis and progression (GARP) study consists of 187 Caucasian sibling pairs and four trios of Dutch origin affected by symptomatic and radiographic OA at multiple sites [31]. Osteoarthritis (OA) is a common degenerative disease of the articulating joints with a considerable, but complex, genetic component. By performing a genome-wide linkage scan and combined linkage and association, the iodothyronine-deiodinase enzyme type 2 (D2) gene (*DIO2*) was identified as an osteoarthritis susceptibility gene. The common coding variant (rs225014; Thr92Ala) in the *DIO2* gene showed significant association. Information about the number of siblings and parents with similar symptoms was available: 30 % of the genotyped affected siblings have no missing (un-genotyped) affected siblings, 30 % have one missing affected sibling. The maximum number of missing affected siblings is 8 (one family). Regarding affected parents, 16 and 60 % of the ASPs had two and one affected un-genotyped parents, respectively. Using this extra information, Callegaro et al. proposed the allele-sharing statistics for genetic linkage analysis to account for the family history, and this considerably increased the evidence of linkage in the surrounding of the *DIO2* susceptibility locus [7].

The question arises whether this strategy could successfully be adapted to a genetic association study. We consider a case–control association study: the 380 cases from the GARP study and the control population from the Leiden Longevity Study described in the previous section [17]. The controls consist of 1671 offspring of nonagenarian siblings (in 420 families) and 744 partners of the offspring. After exclusion of patients with OA and unknown status, 1947 subjects were served as controls.

**Fig. 1:**
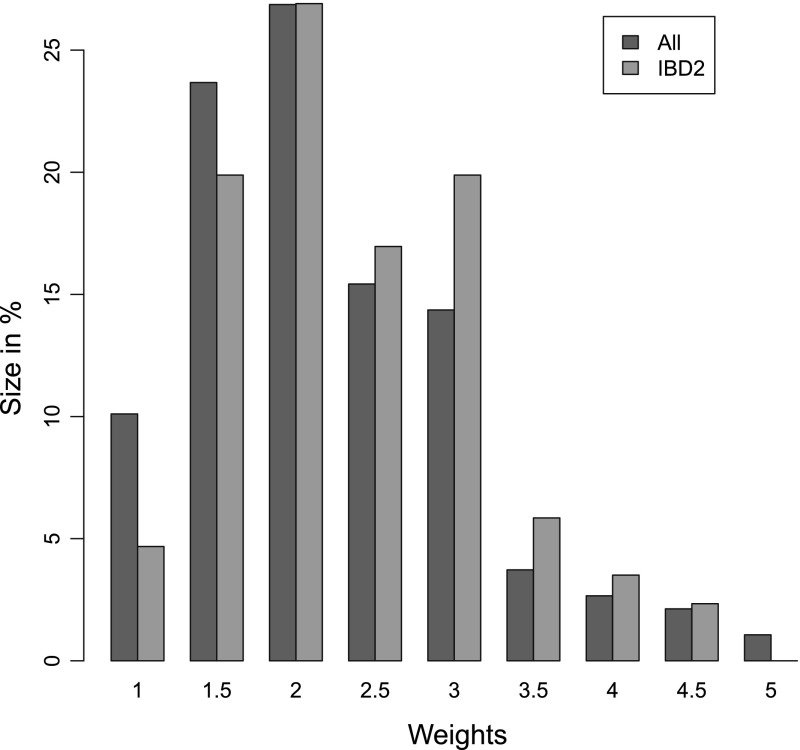
Weights in ST$_\mathrm{fam}$ for all GARP ASPs compared to ASPs with 2 IBD (Color figure online)

Suggestive evidence for linkage was observed on chromosome 14q32.11 (75–95 cM; mean informativity = 0.50, [31]), wherein the genes *DIO2* (78 cM), *FLRT2* (82 cM), and *CALM1* (89 cM) were located. First, the linkage region of 75–95 cM was converted to the physical map, 79147631–95189589 bp, using a build GRCh37 genetic map (ftp://ftp.ncbi.nlm.nih.gov/hapmap/recombination/2011-01_phaseII_B37/). The GWAS data for both the GARP and LLS study were available, and the genotype imputation was performed using IMPUTE2 (http://mathgen.stats.ox.ac.uk/impute/impute_v2.html) based on the reference panel from the February 2012 release of the 1000 Genomes project (ftp://ftp.1000genomes.ebi.ac.uk/) and genome build 37. For the respective linkage region ca. 200K SNPs were available. We performed the association analysis, and the following filtering was employed: MAF in controls ${>}1~\%$, IMPUTE info$>0.4$, and $R_2^T>40~\%$. After filtering out insertions and deletions (indels), the resulting number of the SNPs tested was 57557. For the two study designs—(1) GARP cases ($n=380$) versus controls and (2) GARP cases with 2 IBD ($n= 152$) versus controls—we compared the score test (ST) to the test that includes positive family history (ST$_\mathrm{fam}$). The weights in ST$_\mathrm{fam}$ in the expression (11) of the two samples are compared in Fig. 1. For the population with 2 IBD, larger weights are given corresponding to the increasing family size of affected members (Fig. 1). Using approximate genome-wide significance threshold of $p$ value = 1E$-$05 as in the previous analysis [16], the $p$ values in boldface are the significant SNPs with corresponding test that passed this threshold in Table 6. The SNPs with significant testing were reported, and in this dataset except one SNP, testing that incorporates positive family history gave smaller $p$ values.

**Table 6:** Results of association testing for the linkage region (75–95 cM)

Marker	chr	Position	al1	al2	EAF$^\mathrm{a}$		Info$^\mathrm{b}$	GARP vs. controls			
					Cases	Controls		ST$^\mathrm{c}$	$R^2_T {}^\mathrm{d}$	ST${_\mathrm{fam}}^\mathrm{e}$	$R^2_{T, \mathrm{fam}}$
rs72688979	14	79826540	C	T	0.023	0.012	0.989	7.97E$-$03	59.9	**6.72E** $-$ **08**	42.8
rs148126909	14	79826567	C	T	0.023	0.012	0.989	7.97E$-$03	59.9	**6.73E** $-$ **08**	42.8
rs73337429	14	92866956	T	G	0.067	0.032	0.979	3.31E$-$05	75.3	**4.28E** $-$ **06**	79.2
rs113235844	14	92876680	T	C	0.060	0.026	0.987	**8.53E** $-$ **06**	79.3	1.03E$-$05	79.0
rs113272510	14	92894256	A	G	0.057	0.024	0.985	3.97E$-$06	73.6	**3.52E** $-$ **06**	69.9

Bold numbers indicate approximate genome-wide significance

$^\mathrm{a}$ Frequency of allele 2 (al2)

$^\mathrm{b}$ Imputation quality measure by IMPUTE

$^\mathrm{c}$ Score test of affected siblings and control design

$^\mathrm{d}$ Relative efficiency measure (9) that reflects imputation uncertainty with respect to the association parameter

$^\mathrm{e}$ Score test that incorporates positive family history

Next we revisited the common coding variant, rs225014, in the *DIO2* gene. In [31], joint linkage and association analysis using GARP ASPs was performed to identify SNPs that explain the observed linkage signal by linkage and association modeling in pedigrees (LAMP, http://csg.sph.umich.edu/LAMP) [23]. A significant predisposing association with the C allele of DIO2 SNP rs225014 was obtained ($p$ value = 0.006). Moreover, allele frequencies in sibling pairs sharing two alleles identical by descent (IBD) at the DIO2 locus (indicating those subjects that contribute to the linkage) were compared with allele frequencies of random controls—a random sample of unrelated subjects aged 55–65 years of the Rotterdam study [15]; the C allele of rs225014 was found significantly associated ($p$ value = 0.025). For confirmation and replication in independent UK, Dutch, and Japanese OA studies, significant recessive association of the C-C haplotype of the *DIO2* SNPs rs12885300 and rs225014 with women with advanced symptomatic hip OA was found.

In the current association study as shown in Table 7, the minor allele frequency (MAF) of the cases (0.368) was almost the same as the MAF in the controls (0.364); observe that we have here a different control population, namely from LLS. Additionally, allele frequencies in sibling pairs sharing two alleles identical by descent (IBD) at the DIO2 locus ($n= 152$ in 85 families)—indicating those subjects that contribute to the linkage—were compared to those in controls, and MAF in cases increased to 0.428, which led to a smaller *p* value. For this specific SNP the magnitude of the $p$ values of the two tests was comparable, which might indicate that a larger number of cases are needed to detect association.

**Table 7:** Results of association study for rs225014

Design	*N* cases	*N* controls	MAF cases	MAF controls	ST$^\mathrm{a}$	ST${_\mathrm{fam}}^\mathrm{b}$
(1) GARP vs. controls	380	1947	0.368	0.364	9.96E$-$01	9.08E$-$01
(2) GARP (IBD = 2) vs. controls	152	1947	0.428	0.364	1.08E$-$01	9.90E$-$02

$^\mathrm{a}$ Score test of affected siblings and control design

$^\mathrm{b}$ Score test that incorporates positive family history

## Discussion

When testing for weak genetic effects, besides the efforts to increase sample size, it is desirable to develop statistical methods that more effectively detect an association. Through simulation we showed that sampling genotyped cases from the high-risk families increases the power, even if only one case was sampled. To further increase the efficiency of the study, the multiplex-case and control design was considered, in which genotype frequencies between cases, each of whom was sampled from multiple affected families, and unrelated controls were compared. The primary reason for choosing this design would be that familial cases are enriched for genetic factors and therefore may be more informative for genetic research, particularly in the presence of genetic heterogeneity and phenocopies. For genetic linkage analysis Callegaro et al. proposed allele-sharing statistics using information on family history [7]. In the same spirit, we investigated the use of readily available family information for genetic association within a framework of a score test.

The first issue related to the proposed test is the ascertainment of the cases. The retrospective likelihood is considered to adjust for ascertainment by conditioning on the phenotypes of family members. For a general phenotype, a score statistic to test for an additive effect of a diallelic locus on phenotype is the genotype–phenotype covariance; the score statistic can be conveniently applied in both prospective and retrospective manners [10, 28]. The score test has a flexible structure that allows testing of multiplicative, dominant, and recessive effects of specific genotype features on (disease) phenotype. Testing recessive effect is straightforward in a score test by using different dosage score $(0,0,1)^\top$ opposed to $(0,1,2)^\top$ for testing an additive model.

Secondly, the methods should allow for familial correlations due to sampling-related individuals. The variance of the score statistic can be readily modified to account for familial relationships based on kinship coefficients. To detect recessive effects of a SNP, however, the calculation of correlation coefficient depends on kinship coefficients as well as allele frequency. Another important extension is to construct a test for X-linked SNPs in mixed-sex-related samples. Combining the two statistics obtained by applying an allele-based test to males and females separately is not a valid test for family data. In this work we adapted the score test using X-linked correlation matrices.

Thirdly, to incorporate the extra phenotypic information, the weighting scheme similar to allele-sharing statistics for genetic linkage analysis of [7] is applied to the association testing. When the type of families is uniform in all families—for example each family consists of 2 sibling pairs and 2 additional phenotype information of un-genotyped parents, the weight is uniform and is equivalent to that of the (ordinary) score test. Using various weights other than the value 0 or 1, the proposed score test tends to the continuous weights in that for the quantitative trait.

As shown in Sect. 3 incorporating positive family history in the test statistic appeared to be a powerful strategy. Just by selecting one case from each family with positive family history the power can be increased. This strategy of selecting cases with positive family history might be advantageous, when disease variant allele is rare and residual variance is small. In particular, for detection of recessive effects of rare variants, the increase in the power was remarkable under the ascertainment schemes that required a minimum of two affected individuals in each family. This finding supports previous results that greater power was achieved by sampling cases from multiplex pedigrees [34]. We also have shown improvement of the association results by including the number of affected relatives who were not genotyped in the score statistic through a real data example. Especially, the power of association using rare SNPs can be much improved by adding phenotypic information of un-genotyped family members. Currently the quality of genotype imputation using family data and its impact on the actual analysis is not yet clear [8]. When possible, utilizing additional information of extensive family members from the previous linkage studies is a viable option opposed to imputing un-genotyped family members.

Another benefit of employing our methods would be the use of the weight to pinpoint the extreme families for further investigation. The weights $y$ implemented in the (ordinary) score statistic depend only on the individual’s case–control status, 0 or 1, whereas the weights now can vary depending on the relationship configurations as well as on the phenotype of individuals. Applying these weights to GARP data showed that larger weights were assigned to ASPs with 2 IBD (Fig. 1). Extrapolating this idea, more efficient weights can be constructed as in [17]. For families selected for excessive survival, they computed the family-specific standard mortality ratio’s (SMRs) to describe lifespan distributions of each generation within a family. Instead of discriminating between cases and controls based on these values they can be directly used as weights, which will induce more variabilities in the test statistic. Another extension would be the joint testing of multiple SNPs as described in [18]. Although our test is intended mainly for the multiplex cases, we have shown that this can equally be applied for related controls. A concern regarding the use of family-based samples for conducting case–control association analysis is the potential for population stratification effects. For well-designed studies this should be of minor concern; this effect can be controlled using genomic controls [14]. In addition, it is possible to use principal component analysis to correct for population stratification [32]. Top PCs can be modeled as covariates with the original outcome, and the residual, the result of removing any stratification effect on the outcome under the null, can be used for further analysis. When family structure is more complicated than siblings or parents such as in an isolated founder population, instead of using *naive* estimator of genotype frequency the best linear unbiased estimator [30] can be used in our score test, and the weight incorporating positive family history can be modified in the similar manner [40].

In the Genome-Wide Association Studies (GWAS) era, genotype imputation has become an essential tool. The imputation of genotypes allows investigators to test association at un-genotyped genetic markers and to combine results across studies that rely on different genotyping platforms. Combining the GWAS results for meta-analysis, using family data under strong ascertainment and using case–control data, can be inefficient and difficult. Here, our test can be of great use, since the score statistics from the individual studies can be easily combined. Another aspect of popularity of imputation is that more and more SNPs are imputed using external information of reference haplotypes from the HapMap and 1000 Genomes Project. The ever-increasing number of tests to be performed calls for a tractable flexible approach such as score test.

In conclusion, incorporating positive family history in the test statistic appeared to be a powerful strategy. Especially, the power of association using rare SNPs can be much improved by adding phenotypic information of un-genotyped family members.
